# Comparative analysis of mouse skeletal muscle fibre type composition and contractile responses to calcium channel blocker

**DOI:** 10.1186/1472-6793-5-4

**Published:** 2005-02-14

**Authors:** Satu Mänttäri, Matti Järvilehto

**Affiliations:** 1 Department of Biology, University of Oulu, Oulu, FIN-90014, Finland

## Abstract

**Background:**

In this study, we examined the correlation between excitation-contraction coupling characteristics and skeletal muscle fibre type by (1) localizing the distribution of dihydropyridine receptor (DHPR) protein and (2) comparing the effect of DHPR blocker on muscles with different fibre type composition, in order to better understand the differences between contractile phenotypes of fibres and to explain the contradictory reports to date on the interaction of dihydropyridines with skeletal muscle isoform of DHPR.

**Results:**

Histochemical experiments revealed that fluorophore conjugated dihydropyridines stain selectively the membranes of muscle fibres. The staining was most evident in type IIA fibres. The major fibre type in gluteus and femoris, revealed by mATPase staining, was IIA (45.0 and 38.1 %, respectively). In gastrocnemius the content of IIA fibres was 22.7 %. Contraction forces before and after the addition of blocker for the three muscles investigated were: gluteus 0.075 ± 0.017 N *vs. *0.052 ± 0.011 N, femoris 0.045 ± 0.005 N *vs. *0.033 ± 0.005 N and gastrocnemius 0.089 ± 0.016 N *vs. *0.075 ± 0.014 N, respectively. The attenuation of contraction force proportional to the cross-sectional area of the muscle was significantly (P = 0.023) higher in gluteus (28.3 ± 3.5 %) and femoris (27.6 ± 3.2 %) as compared to gastrocnemius (16.1 ± 2.5 %). However, no significant change in the control measurements was observed ruling out the possibility of fatigue.

**Conclusion:**

The results indicate that the attenuation of the contraction force was largest in muscles with a high percentage of type IIA fibres. This supports our finding that the abundance of dihydropyridine receptors of IIA fibres outnumbers that in the other fibre types. The present data show that the correlation of density of dihydropyridine receptors can be one of the important factors influencing the overall contractile properties of the muscle and for its part explain the contradictory results of previous studies on coupling process.

## Background

One of the features characterizing mammalian skeletal muscle tissue is the structural variability of muscle fibres. The inclusive properties of skeletal muscle lead to functional diversity of muscle fibres, which has been related to differences in relative proportions of membrane structures and to different amounts of contractile proteins [1]. Although the interfibre-type differences are well recognized, very little is known about the characteristics of the process leading from electrical membrane excitation to contraction (E-C coupling) related to different cell types. The first indication of variation in E-C coupling between representatives of different fibre types came from studies using vaseline-gap technique in order to record slow calcium currents and asymmetric charge movement in fast- and slow-twitch muscles [2]. The results suggested functional and structural differences between fast- and slow-twitch mammalian muscles with respect to dihydropyridine receptor (DHPR) density. Recently Goodman et al [3] provided the first evidence about E-C coupling characteristics being related to myosin heavy chain based fibre type and the muscle from which the fibre originated. Our finding about the density of DHPR varying between different fibre types [4] supports the hypothesis that optimum contractile function of skeletal muscle is related to its fibre type composition via differences in E-C coupling.

Voltage-dependent dihydropyridine receptors play an important role in the function of skeletal muscle. As well known, contraction is initiated by a depolarization of the transverse tubular (T-tubular) membrane, which in turn causes calcium to be released from the stores of sarcoplasmic reticulum (SR). The DHPR is located in the T-tubule membrane acting as a voltage sensor. Linked to the ryanodine receptor located in the sarcoplasmic reticulum membrane, this channel protein triggers the intracellular Ca^2+^-release via the ryanodine receptor [5]. The DHPR itself is a heteromultimer composed of 5 subunits; α_1 _(170 kDa), α_2 _(140 kDa), β (55 kDa), γ (33 kDa) and δ (24–33 kDa) [6,7]. The pore forming α_1 _subunit contains the receptor for dihydropyridines (DHPs) and other calcium antagonists. DHPs, such as nifedipine, nitrendipine, and nimodipine, bind to DHP receptor in a specific, saturable and reversible fashion. In addition, receptors show high affinity for DHP ligands. The dissociation constant (*K*_*D*_) for skeletal muscle membranes is usually in the range of 1–10 nM [8]. Dihydropyridines are used clinically as blockers in the treatment of hypertension and coronary heart disease [9].

Besides being the voltage sensor for Ca^2+ ^release needed in E-C coupling, the α_1_-subunit of the molecule functions also as a calcium channel [10] giving rise to a slow inward calcium current [11]. However, DHPR appears to be unimportant as an ion-conducting channel [12]. Firstly, the calcium current is activated too slowly to generate contraction of the fibre. Second, the contraction continues even if the extracellular calcium is removed, and finally, blocking the slow calcium current through the channel does not prevent contraction. On the other hand, Beam et al [5] reported a notable increase in the magnitude of inward calcium current in developing muscle thus implicating an important role of the current in maturation.

In the present study we examined the E-C coupling characteristics of muscles with different dihydropyridine receptor expression defined by fibre type composition. To test the hypothesis that muscles with different cell type compositions, i.e. DHPR quantities, react differently to calcium channel blocker, we measured contraction forces of three muscles before and after adding the blocker solution. We found variation in the activities of muscles depending on the cell type composition of the muscles. The findings confirm the previously observed pattern and elucidate the role of dihydropyridine receptor in muscle cell contraction.

## Results

### Myosin heavy chain analysis and fibre typing

**Figure 1:**
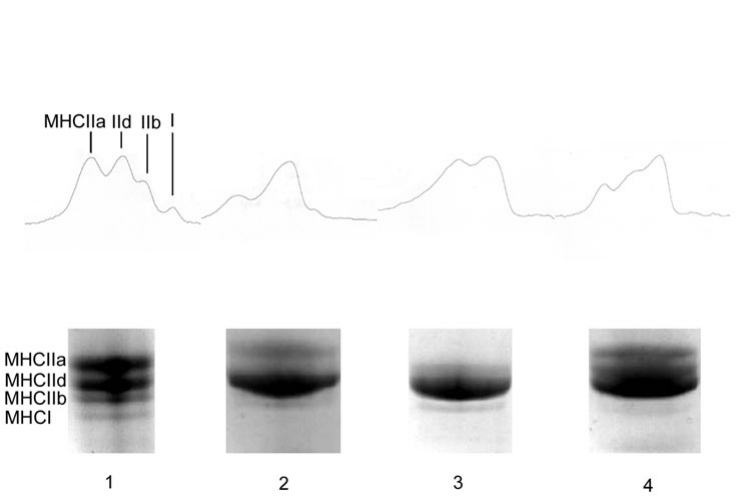
**Electrophoretic separation of myosin heavy chain isoforms. **Myosin isoforms of mouse muscles were compared to those of rat gastrocnemius muscle (lane 1). Densitogram of each lane is shown above the gel. The muscles analysed were m. gastrocnemius (lane 2), m. gluteus (lane 3) and m. rectus femoris (lane 4).

In order to analyse the distribution of cell types in the gluteus maximus (GLU), rectus femoris (RF) and gastrocnemius (GAS) muscles, tissue samples were collected and subjected to SDS-PAGE. The different muscles of mouse contained four myosin heavy chain (MHC) isoforms in the increasing electrophoretic mobility, MHCIIa, MHCIId, MHCIIb and MHCI (Fig. 1) as compared to those of rat gastrocnemius muscle (in this report MHC isoform expressed in fibre is identified by roman numeral and lower case letter, while the fibre type is identified by a roman numeral and a corresponding capital letter according to Hämäläinen and Pette [13]). The distribution of these myosin heavy chain isoforms differed among the three muscles studied (Table 1). In GAS, the major isoform was MHCIId (35.6 %), expressed together with smaller amounts of IIb (28.1 %). On the other hand, the quantity of MHCIIa was smaller (20.6 %). In contrast to GAS, both GLU and RF were characterized by higher contents of MHCIIa (26.6 % and 27.7 %, respectively), together with MHCd (34.3 %; 23.7 %) and lower amount of MHCIIb (15.9 %; 24.3 %). All the muscles contained also portions of MHCI (GAS: 15.7 %, GLU: 23.2 %, RF: 24.3 %). A statistically significant difference (P < 0.05) was found in the portion of IIb between GAS and GLU as well as in IId between GAS and RF. Four fibre types were also identified in muscle sections stained with mATPase (Table 1). The major fibre type in GAS was IID (51.5 %). However, in contrast to the results from SDS-PAGE, IIA fibre type (22.7 %) outnumbered the IIB (18.2 %). The most frequent fibre type in both GLU and RF was IIA (45.0 %; 38.1 %), followed by IIB (31.1 %) in GLU and IID (21.6 %) in RF. There was a statistically significant difference (P < 0.05) in the portion of IIA between GLU and RF. In addition, between GAS and RF a significant difference was found in the proportion of I (P < 0.05) and IID (P < 0.01). Between GAS and GLU a significant difference was found in proportion of IIA (P < 0.05) and IID (P < 0.01). To summarise, the significant difference in proportion of IIA between GAS and RF/GLU is particularly relevant to the present study.

### DHPR expression and correlation to fibre types

**Figure 2:**
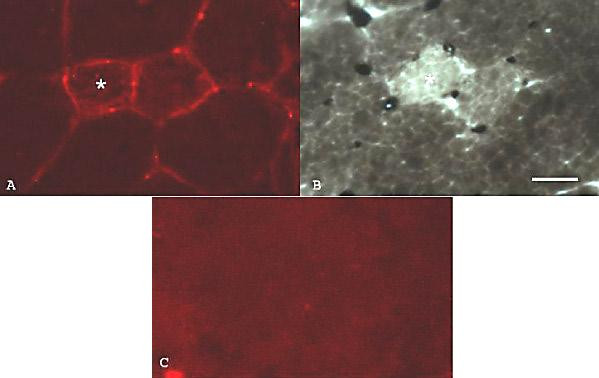
**Characterization of fibre type specificity of dihydropyridine receptor **Two subsequent sections of mouse musculus gastrocnemius were assayed for DHPR localization by fluorescence staining (A) and for fibre type analysis by staining for mATPase (B). After preincubation in pH 4.6, type IIA displays a relatively low acid stability and is stained most lightly (*). The corresponding fibre in (A) is also recognized by fluorophore conjugated DHP blocker. The fibre on the right of the asterisked fibre shows also fluorescence. The intensity is, however, somewhat lower than in the fibre next to it and originates probably from the special fibre type (hybrid, type IIDA). (C) A control sample preincubated with nifedipine. Bar 20 μm.

We have previously shown that the density of dihydropyridine receptors (L-type calcium channels) in T-tubule membranes increases markedly during the postnatal development of mouse skeletal muscle [4]. Furthermore, the findings showed that the fast oxidative glycolytic (FOG) fibre type has the most evident appearance of DHPRs. Fig. 2A shows localization of DHPR in mouse RF muscle. DHPR is strongly expressed in some muscle fibres whereas weakly expressed at the membrane of others. The corresponding control slide, preincubated with nifedipine resulted in a loss of staining (Fig. 2C). To correlate the DHPR expression with muscle fibre typing, histochemical experiments were performed as described. According to the staining intensity, IIA fibre type corresponds to the muscle fibres that strongly express DHPRs (Fig. 2B).

### Force measurements

**Figure 3:**
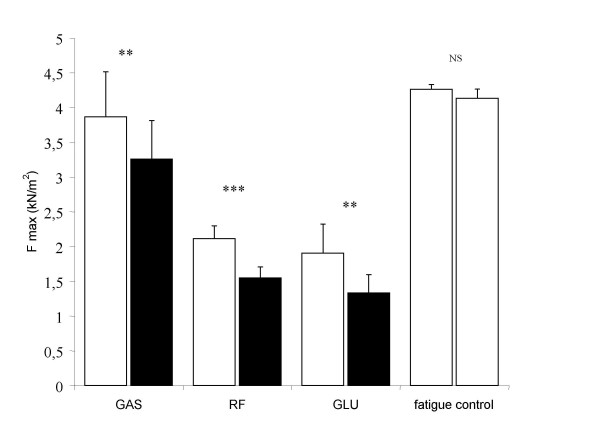
**Effect of nifedipine on contraction force. **Maximum contraction forces of GAS, RF and GLU in proportion to the cross-sectional area of the muscle (white), and the average responses to 1 μM nifedipine solution (grey) are shown. The fatigue control was performed with GAS, RF and GLU in physiological salt solution devoid of nifedipine in order to control the procedure and to rule out the possibility of fatigue. The statistical significance of differences between means of contraction forces before and after the addition of nifedipine solution is shown by levels (*, P < 0.05; ** P < 0.01, paired samples t-test).

**Figure 4:**
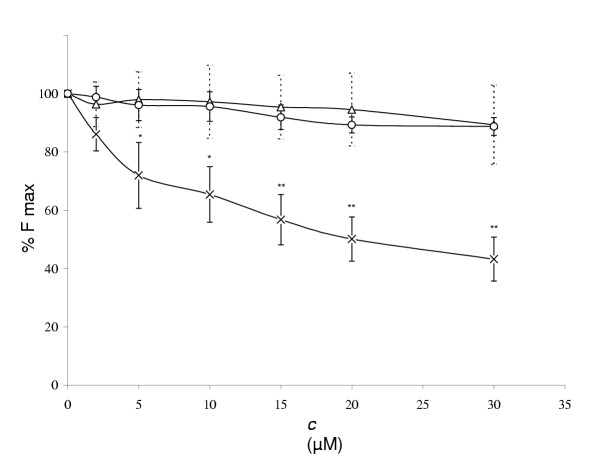
**A force-concentration curve for GAS (Δ) and GLU (×) **GLU was also used as a saline standard (○) in order to rule out the possibility of fatigue. The x-axis displays the increase of nifedipine concentration and the y-axis shows the relative force response of the muscles in % of the maximum initial value. Values are given as means ± SE (dashed lines for GAS). The significance of the decrement of contraction force in proportion to the cross-sectional area of the muscle is shown by levels (*, P < 0.05; ** P < 0.01, paired samples t-test) in GLU at nifedipine concentration of 5 μM onwards. N = 6 in each measurement.

GLU, RF and GAS muscles were weighed and the length and diameter were measured. Table 2 shows some morphological details for the muscles studied. To reveal the function of L-type calcium channels in muscles with different kinds of cell type distributions, the single twitch forces of the muscles were measured before and after the addition of a specific channel blocker nifedipine. Maximum contraction forces in proportion to the cross-sectional area of the muscle, and the average responses to 1 μM nifedipine solution are shown in Fig. 3. The force production of GAS (0.089 ± 0.016 N) was considerably higher in comparison to the ones of GLU (0.075 ± 0.017 N) and RF (0.045 ± 0.005 N). The effect of nifedipine on contractile force was inhibiting in all the muscles studied. The *in vitro *nifedipine effect on the muscle force production was determined as the percentage of the remaining channel mediated contraction force. The weakening of contraction force proportional to the cross-sectional areas of the muscles was significantly (P = 0.023) higher in GLU (28.3 ± 3.5 %) and RF (27.6 ± 3.2 %) in comparison to GAS (16.1 ± 2.5 %). Experiments performed in increasing concentrations of nifedipine resulted in a clear dose-response curve (Fig. 4) and revealed a decreasing force output as the blocker concentration increased. Furthermore, the decrease was significantly smaller in GAS (P < 0.05 at nifedipine concentration of 5 μM), in comparison to the decrease in GLU.

## Discussion

The cell type composition of a muscle is one of the features characterizing the function of the muscle. Type I fibres are most prevalent in muscles involved in the maintenance of posture, whereas type II fibres are used for movements which require high power output. Many vertebrate locomotor muscles are composed of a mixture of fibre types. Additionally, one muscle fibre may contain several myosin heavy chain types indicating heterogeneity at fibril level [14]. The m. gastrocnemius (whole) used in this study has a high type IIB content whereas both m. rectus femoris and m. gluteus maximus are predominantly composed of types IIA and IID. The results from myosin heavy chain analysis and fibre typing are somewhat different. The identification of four different fibre types using histochemical methods is difficult because of the presence of several types of myosin heavy chains within one single fibre. Therefore, the MHC analysis is more qualitative. The correlation of high density of DHPRs with IIA fibres was stated on the basis of ATPase activity. This was previously also shown by statistical analysis where fibre size was additionally taken into account [4].

Nifedipine was used as an antagonist in order to specifically block the dihydropyridine receptors. Nifedipine is a dihydropyridine derivative that binds in a specific, stereoselective and saturable fashion to DHPR. The selective calcium channel inhibitor prevents the influx of extracellular calcium through the L-type calcium channel [15] and also has a clear effect on the contraction activity of the skeletal muscle fibre. The effects of nifedipine on depolarization-induced force responses are inhibitory and dose dependent [16]. As the half life of the blocker is 2 – 6 h, there is enough time to perform reliable measurements.

By blocking the channel with nifedipine, a clear selective decrease in the contraction force of the different muscles studied was observed (Fig. 3). The inhibition percentages of RF and GLU were, however, significantly higher as compared to that of GAS. This data indicates that the same concentration of blocker causes a different response between different muscles. A similar variability is noted when compared to the cell type composition of the muscles. The muscles with high IIA fibre type content have strongly reduced contraction force as a result of addition of a calcium channel blocker. On the other hand, the effect of a blocker on the muscle with a lower IIA type content is much weaker. The findings are attributable to the varying densities of dihydropyridine receptors in muscles. In GAS, the amount of DHPRs is reduced, likely due to the low IIA fibre type content. Thus the blocking effect of a receptor antagonist is weaker as compared to the muscles with a higher IIA content. Furthermore, the response of GAS and GLU to the gradual saturation of DHPRs with nifedipine molecules is different (Fig. 4). Consistently, the inhibition percentage of the contraction force of GAS decreased less as compared to GLU. In addition, a constant level of the contraction force was reached sooner in GAS indicating a complete saturation of the receptors available.

There are several examples of the divergent behaviour of the dihydropyridine receptor in skeletal muscle. First, dihydropyridines are shown to have both stimulatory [17] and inhibitory [18] effects on excitation-contraction coupling. Furthermore, unlike in cardiac muscle, calcium release from SR does not require inward current, and as yet is induced by blockade of dihydropyridine receptors [19]. However, it is clear that DHPRs are essential in excitation-contraction coupling since animals with *mdg/mdg *mutation, which results in a lack of receptor proteins, die at birth because of paralysis of the respiratory muscles [20]. On the other hand, when the α_1_-subunit is introduced into the nuclei of the dysgenic myotubes, some cells contract upon an electrical stimulation. Despite of this, the recovered influx of calcium ions is not necessary for the contraction since the cells are contracting also even if the current is blocked with cadmium. Hence calcium antagonist drugs seem to have very few pharmacologically relevant actions on skeletal muscle as observed also by Walsh et al [21]. The role of inward current is still an open question although it has been suggested that the current is needed to maintain the calcium stores inside the cell [5] or for the conditions of activeness of the voltage sensor [22].

In the present study we report one plausible explanation for the large variation of the results observed in the behaviour of the L-type calcium channels in skeletal muscle. The uneven density distribution of dihydropyridine receptors indicates a difference in E-C coupling machinery between muscle fibre types. In this study, we showed that the difference is also detectable in the contraction forces between muscles of different cell type composition. Although nifedipine specifically blocks the dihydropyridine receptor, there could also be other differences in addition to the density of receptors between the muscles causing the difference in the contraction forces. A larger number of different muscles and developmental stages might clarify the differences in the present results.

Goodman et al [3] provided the first evidence that E-C coupling characteristics are related to fibre types based on myosin heavy chain. By measuring depolarization-induced force responses of skinned single fibres of rat, they concluded that the optimum force production of a skeletal muscle is related to its MHC isoform composition. Although we found no published data on the E-C coupling phenotype of MHC IIa isoform, the results from previous studies suggest that the parameters describing force and velocity properties of a single muscle fibre are significantly higher in the fast, type IIA and IIB, fibres than in slow, type I fibres.

## Conclusions

Taken together, our results obtained from three different muscles confirmed that the expression of dihydropyridine receptor is a fibre type specific character. In addition, the present data indicate that the density of DHPRs is one important factor influencing the overall contractile properties of the muscle. Furthermore, the results of our experiments point out that when determining the physiological relevance of DHPRs, it is necessary to compare histochemistry and protein analysis to relevant functional properties such as contraction of the muscle. Moreover, it is noted, that differences between developing and adult type of muscle cells may emerge due to the differentiation of phenotypes of muscle fibres. In the future it might be useful to examine the correlation from a larger number of different muscles, and from muscles of different developmental stage.

## Methods

### Electrophoretic separation of myosin heavy chain isoforms

M. rectus femoris (N = 6), m. gastrocnemius (N = 6) and m. gluteus maximus (N = 6) from adult mice (strain CD-1) were removed after the animals were killed by paracervical dislocation in accordance with the Animal Ethics Committee of the University of Oulu (licence no. 046/03). The muscles were homogenized in 6 vol of homogenization buffer [62.5 mM Tris-HCl, pH 6.8] and boiled in sample buffer as previously described for 5 min at a final protein concentration of 0.5 mg ml^-1 ^[23]. Total protein was assayed according to the method of Bradford [24]. Myosin heavy chain isoforms were separated by gradient (5–8 %) sodiumdodecylsulfate polyacrylamide gel electrophoresis performed at 5°C for 23 h (120 V constant voltage). The gels were stained with Coomassie Brilliant Blue. The stained gels were scanned and the separated protein bands were analysed with FluorS MultiImager program (Bio-Rad, USA).

### Fibre typing

Serial cross-sections, 8 μm thick, were cut on a cryostat microtome at -25°C, mounted on cover slips and stained for myosinATPase (mATPase) with acid preincubation according to Hämäläinen and Pette [25]. The sections were preincubated at room temperature for 7 min in sodium acetate (54.3 mM) – sodium barbital (32.6 mM) solution adjusted with HCl to pH 4.6. After washing with CaCl_2 _(18 mM) and Tris-HCl (199 mM) the sections were incubated in substrate solution (4.5 mM ATP, 19.5 mM CaCl_2_, 116 mM 2-amino-2-methyl-1-propanol; pH 9.4) at room temperature for 45 min. After incubations in 11 mM CaCl_2_, 2% CoCl_2_, and 10 mM sodium barbital, the colour was developed in 2% (v/v) ammonium sulphide for 45 s. After washing with distilled water, the sections were dehydrated in ethanol, cleared in xylene and mounted with DPX. An alkaline preincubation in a solution containing 34 mM 2-amino-2-methyl-1-propanol, 120 mM CaCl_2_, adjusted to pH 10.3 with HCl was also used as previously described by Guth and Samaha [26]. After preincubation the sections were processed as above. The percentage of each fibre type in different muscles was calculated with the use of the LSM 5 PASCAL software 3.2 (Leo, Germany) for analyzing images.

### Fluorescence staining

For fluorescence labelling, RF muscle from mouse was cut into 8 μm cryostat sections, dried for 30 min and treated as previously described by Mänttäri et al [4]. The concentration of the high affinity (-)-enantiomer of dihydropyridine was 20 nM. Control samples were preincubated with 10 μM nifedipine in the phosphate buffer for 10 min prior to the addition of dihydropyridine conjugate.

### Force measurements

Adult mice (weighing 23 – 45 g) were killed by paracervical dislocation, and the pelvic region and right hind limb were skinned. The muscle of interest (m. rectus femoris, m. gastrocnemius or m. gluteus maximus) was dissected out and suspended between a fixed clamp at the base of an organ bath and a Grass FTO3C (USA) force-displacement transducer. Muscles were maintained in an oxygenated (95% O_2 _– 5% CO_2_) physiological saline solution (+37°C, pH 7.4) containing 137.0 mM NaCl, 2.7 mM KCl, 1.0 mM MgSO_4_· 7H_2_O, 1.8 mM CaCl_2_, 0.4 mM NaH_2_PO_4_, 12 mM NaHCO_3 _and 5.5 mM glucose. After an equilibration period of 2 min, the muscles were supramaximally stimulated using steel electrode, pulses of 1 ms duration at 80 V (model S44, Grass Instrument Co.) and muscle length selected to elicit maximal twitch force. The maximum force of the muscle was measured in series of three stimulus impulses with one minute equilibration time between the stimulations. Recordings of transducer output were A/D converted and collected on a computer at a sample frequency of 1000 Hz.

In order to examine the role of a specific, L-type calcium channel blocker, the muscles first measured in saline solution were mounted in a bath containing 1 μM nifedipine [1,4-dihydro-2,6-dimethyl-4-(2-nitrophenyl)-3,5-pyridinedicarboxylic acid dimethyl ester], equilibrated for 2 min, and assessed as described above i.e. the muscle was a control in itself. In addition, series of measurements were made with m. gastrocnemius (N = 6) and m. gluteus (N = 6) using nifedipine concentration range of 2 – 30 μM. The concentration of the drug was progressively increased by adding the nifedipine solution to the saline solution bath. During the experiment, the nifedipine solutions were maintained in dark to prevent photo-bleaching. To exclude the possibility of fatigue caused by successive stimulations, the protocol control measurement was performed similarly devoid of nifedipine.

### Statistical analysis

The statistical significance of differences between means of contraction forces before and after the addition of calcium channel blocker was evaluated by paired samples t-test. The difference in parameters between the different types of muscle were analysed with independent samples t-test for equality of means. A P value of < 0.05 was accepted as indicative of a significant difference between the two sets of observations. The significance of differences between the muscles was evaluated with one way analysis of variance (normality test passed). The significant difference was stated as mentioned above. The values are presented as the means ± SE. All the statistical analyses were performed with the SPSS for Windows software.

## List of abbreviations used

DHP; dihydropyridine

DHPR; dihydropyridine receptor

E-C coupling; excitation-contraction coupling

FOG; fast oxidative glycolytic

GAS; *musculus gastrocnemius*

GLU; *musculus gluteus maximus*

mATPase; myosin adenosine triphosphatase

MHC; myosin heavy chain

RF; *musculus rectus femoris*

SDS-PAGE; sodium dodecyl sulphate polyacrylamide gel electrophoresis

SR; sarcoplasmatic reticulum

T-tubule; transverse tubule

IIa, IIb, IId; myosin heavy chain type

IIA, IIB, IID; fibre type

## Authors' contributions

SM carried out most of the experiments, assisted in interpretation of results, and participated in writing the manuscript. MJ conceived of the study, participated in its design and coordination, and participated in writing the manuscript.

## Figures and Tables

**Table 1:** The proportion of fibre types in different muscles. The fibre type composition of m. rectus femoris, m. gastrocnemius and m. gluteus maximus were determined by SDS-PAGE based on electrophoretic separation of myosin heavy chain isoforms, and staining for myofibrillar ATPase after acid and alkaline preincubation. IIa, IIb, IId; myosin heavy chain types, IIA, IIB, IID; fibre types. Statistical significance of the differences between the corresponding groups (I vs. I, IIa vs. IIA etc.) from the two methods used: NS non significant; * *P *< 0.05; ** *P *< 0.01; *** *P *< 0.001.

	**muscle fibre populations % (SE)**		
	
**SDS-PAGE**	**m. rectus femoris**	**m. gastrocnemius**	**m. gluteus maximus**
I	24.3 (3.0)	15.7 (4.6)	23.2 (3.0)
IIa	27.7 (1.1)	20.6 (3.2)	26.6 (2.9)
IIb	24.3 (2.3)	28.1 (3.1)	15.9 (1.4)
IId	23.7 (1.7)	35.6 (2.2)	34.3 (1.3)


total	100.0	100.0	100.0
			
**mATPase**			

I	21.1 (2.3) ^NS^	7.6 (1.3) **	8.0 (2.6) **
IIA	38.1 (1.5) ***	22.7 (3.8) ^NS^	45.0 (0.8) ***
IIB	19.2 (2.3) *	18.2 (1.8) **	31.1 (3.6) **
IID	21.6 (1.1) ^NS^	51.5 (3.1) ***	15.9 (1.6) ***

**total**	100.0	100.0	100.0

**Table 2:** A selection of morphological features of different muscles.

		mass		size	
		
muscle	N	body (g)	muscle (g)	length (mm)	diameter (mm)
m. rectus femoris (SE)	8	32.13 (1.64)	0.12 (0.01)	11.10 (0.23)	5.20 (0.20)
m. gastrocnemius (SE)	12	31.25 (2.18)	0.15 (0.01)	11.41 (0.31)	5.95 (0.19)
m. gluteus maximus (SE)	13	27.50 (1.51)	0.11 (0.01)	12.70 (0.30)	6.61 (0.24)
